# The Relationship Between Inflammation and Central Nervous System in Multiple Sclerosis

**DOI:** 10.1002/acn3.70231

**Published:** 2025-10-24

**Authors:** Gamze Ansen, Ali Behram Salar, Abdulkadir Ermis, Erkingul Birday, Lutfu Hanoglu, Bayram Ufuk Sakul

**Affiliations:** ^1^ Department of Anatomy, Faculty of Medicine Istanbul Medipol University Istanbul Turkey; ^2^ Department of Physiology, Faculty of Medicine Istanbul Medipol University Istanbul Turkey; ^3^ Department of Neurology, Faculty of Medicine Istanbul Medipol University Istanbul Turkey

**Keywords:** functional connectivity, immune system, relapsing–remitting MS, resting‐state fMRI

## Abstract

**Aim:**

Multiple sclerosis is an autoimmune demyelination disease that is seen especially in the young population and has a progressive course, causing motor, sensory, and cognitive deficits. In the literature, the pathogenesis of MS disease and the interconnection between the immune and central nervous system in the disease have not been fully revealed. Recent studies indicate that gray matter damage, as well as white matter lesions, are frequently seen in MS patients. Based on this background, the present study aimed to explore whether relapsing–remitting MS patients in the attack phase demonstrate different patterns of functional connectivity compared to those in a stable phase.

**Material and Method:**

For this purpose, resting‐state fMRI findings of the attack (*n* = 5) and stable (*n* = 14) groups were examined.

**Results:**

Compared to stable patients, the attack group appeared to show increased functional connectivity in several gray matter structures, including the left fusiform, posterior cingulate, orbitofrontal cortex, left supramarginal gyrus, thalamus, and precuneus.

**Conclusion:**

The findings indicate that patients in the inflammatory phase may exhibit increased activation in distinct gray matter regions relative to those not in the attack phase. This pattern might reflect the development of compensatory functional connections aimed at limiting potential clinical damage during relapse. Moreover, considering the diverse roles of these regions, their involvement could hypothetically be linked to immune‐related processes, a possibility that warrants further investigation in larger cohorts.

## 1 Introduction

The immune system is a mechanism formed by the cells and molecules with unique properties against infection and inflammation that may occur in the organism. It reveals rapid, specific, and protective responses against microorganisms. This defense mechanism prevents or limits infections that may be caused by pathogenic microorganisms. The primary immune response begins when a foreign antigen interacts with an antigen‐specific lymphocyte under appropriate conditions. This response usually involves the production of antigen‐specific antibodies and the differentiation of effector T‐lymphocytes. As soon as an antigen enters the body, an immunological memory is formed in the organism whose immunity has developed. The first infection response developed against the microorganism initiates the immunity and prepares the body against recurring situations. Another feature of the immune system is that it can distinguish between foreign antigens coming from outside the body and antigens released by the body's own tissues [1].

Specialized T‐cells are regulatory cells that function to suppress the immune response that may occur against the body's own antigens. This situation, in which the immune system does not damage its own antigens, is called “immunological tolerance.” In case of any problem or mutation in the cells that provide immunological tolerance, the immune system creates a response against its own antigens and, as a result, autoimmune diseases occur. Systemic lupus erythematosus, rheumatoid arthritis, and multiple sclerosis (MS) are very important autoimmune diseases [1]. Although autoimmune diseases are relatively rare, their effects on mortality and morbidity can be considerable. The exact mechanisms underlying autoimmunity remain incompletely understood [2, 3].

The brain receives and evaluates information from the peripheral immune system in order to make appropriate changes regarding the immunological status of the body. This is called “immunoception” [4]. For instance, in the cases of sepsis and severe acute inflammation, which can cause serious damage to the body, changes in feeding behavior, metabolism, and fever are initiated by the brain [5]. Recent studies have shown that the pathogenesis of inflammation is regulated by a two‐way interaction between the central nervous system (CNS) and the immune system [6]. The immune system informs the CNS about inflammation in the body and ensures that it is controlled through neuronal and neuroendocrine pathways. Some evidence indicates that there are six hypothalamic nuclei/regions that have extensive communication with other brain regions such as the insula, anterior cingulate, and frontoparietal cortex associated with inflammation in humans [7]. It has been reported that the insular cortex stores information about peripheral immune responses and, in the case of inflammation, elicits appropriate responses in the body through its connections to various regions of the brain [8]. Furthermore, brain regions such as the hippocampus and amygdala are also responsible for storing information about the inflammatory condition and generating an immune response when necessary [9].

Multiple sclerosis (MS) is generally defined as an autoimmune disease caused by organ‐specific T‐cells [10]. It is the most common disability affecting young adults without a history of trauma [11]. The incidence and prevalence of MS are increasing in both developed and developing countries, and the underlying cause is not fully known [12]. The disease is monitored in two stages: early inflammation, which is responsible for recurrence and recovery phase, and the neurodegeneration stage, which occurs in the late period without causing recurrent progression [10]. The disease may be mono or polysymptomatic depending on the location of involvement and usually presents with clinical symptoms such as optic neuritis, brainstem and spinal cord syndromes, and some cortical region involvement. In addition to clinical evaluations, MS is diagnosed by serological tests (oligoclonal immunoglobulin G (IgG) bands and neurofilament light chain (NfL)) and magnetic resonance imaging (MRI) to eliminate other pathologies [13]. The radiological method used to determine the presence, location, number, and size of lesions in the brain and medulla spinalis in MS is T2‐weighted conventional and contrast‐enhanced T1‐weighted MRI [14]. The pathological differential criterion of MS is perivenular inflammatory lesions that cause demyelinating plaques in MRI [15]. Oligodendrocyte damage and demyelination occur as a result of inflammation. In the early stages of the disease, axons are preserved. Irreversible axonal damage occurs with disease progression [16]. Although axonal (white matter) damage has long been emphasized, cortical (gray matter) atrophy has also been increasingly recognized, and in some studies, it appears to progress more rapidly, independent of white matter changes [17]. It is thought that damage to cortical regions also explains the cognitive impairments seen in the course of the disease [18].

Various guidelines have been developed over the years to diagnose and monitor MS. The first McDonald criterion was published in 2001, and many versions have been developed over the years. The latest McDonald criteria were published in 2017 and brought together various items in the diagnosis of MS. This version focuses on oligoclonal band formation in addition to clinical findings and MRI results [19].

According to the symptoms and process of the disease, MS patients are classified in four categories as: relapsing–remitting MS, primary progressive MS, secondary progressive MS, progressive‐relapsing MS. In most relapsing–remitting MS (RRMS) patients, the group included in our study, disease progresses in the early stages with a period in which symptoms due to white matter inflammation and lesions in the nervous system appear (relapsing) and a period in which these symptoms disappear or decrease (remitting) [20]. Symptoms in patients with RRMS have been reported to relapse due to increased myelin‐specific T‐cell responses resulting from systemic infections [21, 22].

Functional magnetic resonance imaging (fMRI) technique has been frequently used in the field of cognitive neuroscience in recent years. This imaging technique is a noninvasive method that can measure neuronal activity in the human brain in resting‐state and task‐based forms [23]. Since changes in functional connections between brain regions are observed in MS, the resting state fMRI method is used to understand the functional mapping of the brain and changes in the clinical course of the disease. Recent fMRI studies have proposed that compensatory functional reorganization may occur in MS patients during recovery phases, and that these new connections could potentially help reduce clinical symptoms [24].

Therefore, the aim of our study was to explore how the inflammatory process in MS is reflected in the CNS. Specifically, we compared resting‐state fMRI data from RRMS patients experiencing an attack period with those from patients in a non‐attack period.

## 2 Material and Method

### 2.1 Patient Criteria and Evaluation

This study was conducted in collaboration with the Department of Anatomy, Physiology and Neurology of Istanbul Medipol University Faculty of Medicine, with the approval of the Istanbul Medipol University Non‐Interventional Clinical Research Ethics Committee. Participants were selected from patients who applied to the Neurology Department Polyclinic at Istanbul Medipol Mega University Hospital. A total of 19 patients diagnosed with RRMS, who met the inclusion criteria and were divided into two groups, 5 in the attack period (three women, two men) and 14 in the stable period (attack‐free period) (eight women, six men), were included in this study. As this study was designed prospectively, all participants were informed about the study procedures and signed written informed consent.

Patients were evaluated by a neurologist, and those who met the appropriate criteria were included in the study.

Inclusion criteria:

1. Adult patients over 18 years of age
2. Attack RRMS group: Patients meeting the 2017 McDonald criteria for RRMS, who had experienced a clinically defined new attack (motor or sensory symptoms) within the last week, and who demonstrated at least one active contrast‐enhancing lesion in the CNS.
3. Stable RRMS group: Patients meeting the 2017 McDonald criteria for RRMS, who presented for routine follow‐up without describing any new clinical attacks and who did not have active contrast‐enhancing lesions on CNS MRI.
4. Patients with early‐stage MS and mild disability, with Expanded Disability Status Scale (EDSS) scores between 1.5 and 3.5 in both groups.
5. Stable general medical condition

Exclusion criteria:

1. Progressive MS patients
2. Patients with newly developed structural brain lesions (e.g., tumor, hemorrhage)
3. Patients with systemic infection, cancer, or severe renal, hepatic, cardiac, or pulmonary failure associated with leukocytosis or elevated acute phase reactants (CRP) in blood tests
4. Patients with moderate to advanced stage MS (EDSS > 3.5)
5. Unstable general medical condition

### 2.2 Acquisition of fMRI Data

Anatomical and functional MRI data were obtained with a Philips Achieva 3 Tesla scanner (Philips Medical Systems, Best, the Netherlands). T1‐ and T2‐weighted anatomical images were acquired as 190 slices with a resolution of 1 × 1 × 1 mm, and functional images were acquired as 300 volumes with a resolution of 3 × 3 × 3 mm (TR/TE: 2300/30 ms). The total scan time was approximately 40 min. To assess resting‐state activity, participants were instructed to remain still with their eyes open and fixated on a central point.

### 2.3 Analysis of fMRI Data

**FIGURE 1:**
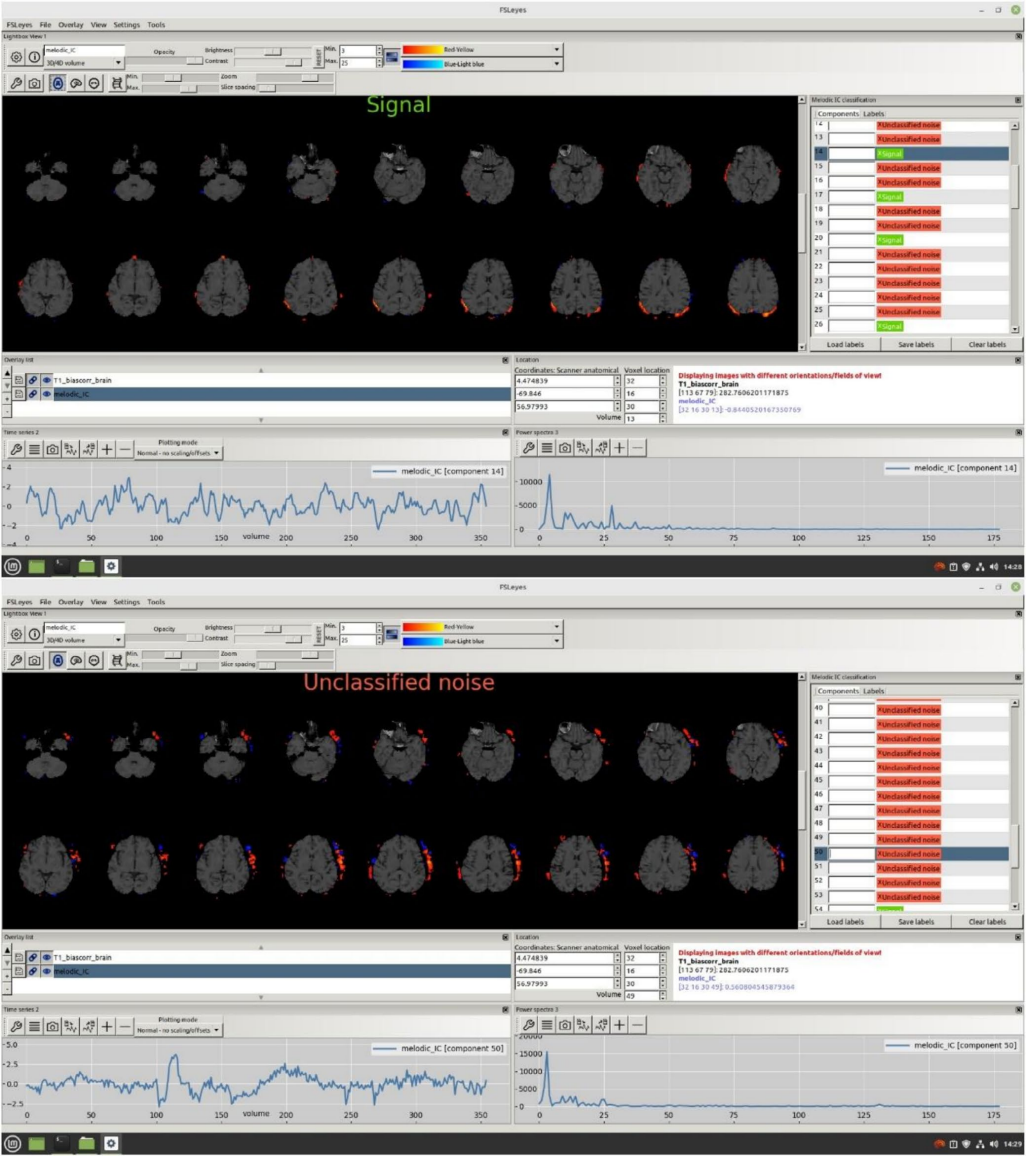
An example of axial section views, time series and power spectrum graphs of components marked as artifacts and signals, visualized through FSLeyes software.

Preprocessing of participants' MRI data was performed using the FSL 6.0 (FMRIB Software Library v6.0) software package within the Linux 18.3 operating system [25, 26]. The anatomical (T1‐weighted) and functional data of each participant were first converted from the raw DICOM format obtained from the MRI device to the NIFTI (nii.gz) format with the help of the software called dcm2niix. Anatomical data converted to standard NIFTI format were then preprocessed with the fsl_anat package script, which is part of the FSL software package. With the help of this package script, bias‐field corrections were made, brain tissue was separated from surrounding tissues, and each participant's MRI data were fitted to the MNI‐152 standard plane linearly and nonlinearly with FLIRT (FMRIB's Linear Image Registration Tool) and FNIRT (FMRIB's Non‐Linear Image Registration Tool) [27, 28]. FEAT visual interface was used in the preprocessing of functional data. With this interface, the possible distortions caused by the movements of the participants during the shooting were corrected with the MCFLIRT system in the FSL package, the brain tissue was separated, the functional data of the participants were matched with their own high‐resolution anatomical data and the MNI‐152 standard plane, and MELODIC (Multivariate Exploratory Linear Optimized Decomposition into Independent Components) and ICA (Independent Component Analysis) independent component analysis were performed [28, 29]. The time series and power spectrum graph of the independent components obtained as a result of ICA were examined and the components that could not be a signal were manually marked with FSLeyes. These components, which were later determined to be non‐signal, were removed from the functional data using the fsl_regfilt script (Figure 1).

### 2.4 Group Analysis of fMRI Data

**FIGURE 2:**
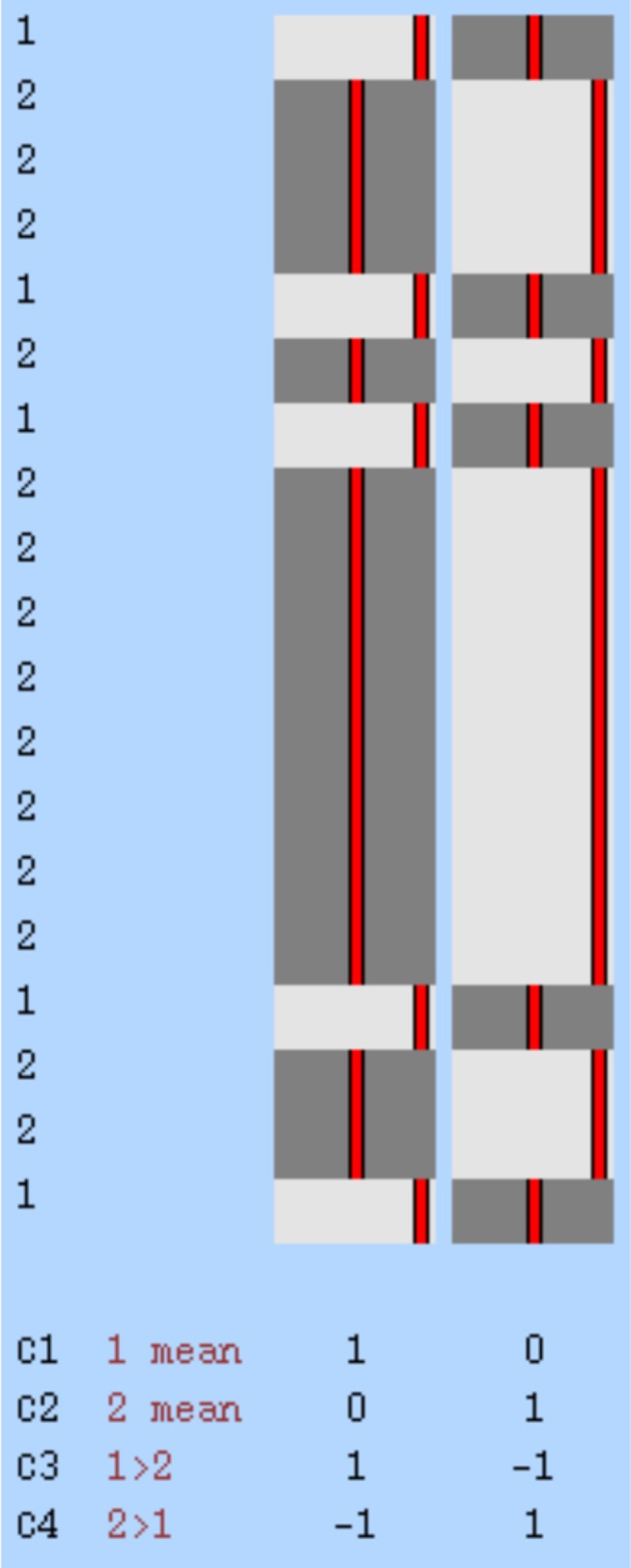
Visual representation of the design matrix created for comparison between groups.

The MRI data, whose preprocessing processes were completed, were classified according to the groups in which the participants were located. In the analysis to reveal the activation differences in the two groups in our study, spatial maps were created with the help of FSL's MELODIC tool (Group ICA) [29]. ICA was performed by adding the participants' data end‐to‐end in time. Twenty independent component maps were created corresponding to the activation areas from all participants in the groups. The design matrix was created with the General Linear Model (GLM), and a mechanism was created to perform group analyses. As a result, four contrasts were determined as given below (Figure 2).

**C1:** Mean activation in MS patients during attack.

**C2:** Mean activation in MS patients during stable phase.

**C3:** Components showing higher activation in attack compared to stable phase.

**C4:** Components showing higher activation in stable compared to attack phase.

For the activation comparison between the two groups, the preprocessed MRI data of each patient, the 20‐component group independent component map, and the design matrix were used as input. The dual_regression script belonging to the FSL software package was used to use these inputs [29, 30]. In the first stage, time series were generated for each component obtained as a result of group ICA from the participants' own functional MRI data. These time series were used as regressors in the second stage, and spatial maps were created for each group of ICA components for each participant. In the third stage, the areas with activation differences between the two groups were identified by using these spatial maps and comparing them statistically with 5000 nonparametric permutations. In order to determine the anatomical structures on the brain structures where the areas with different activation correspond, Harvard‐Oxford cortical and subcortical atlases in the FSLeyes program were used.

## 3 Results

### 3.1 Demographic Information of Participants

Statistical analyses for demographic information (age and gender) of the groups included in the study were performed using the SPSS 18.00 package program. Data were expressed as mean, standard deviation, and percentage (%). *p* < 0.05 was considered statistically significant.

The age and gender distribution of the people included in the study according to the groups is given in Tables 1 and 2, respectively.

**TABLE 1:** The ages of the groups are distributed homogeneously.

Group	*N*	Min age	Max age	Sum	Mean	Std. deviation
Attack MS	5	21.00	33.00	146.00	29.2000	4.91935
Stable MS	14	21.00	49.00	444.00	31.7143	9.14354

**TABLE 2:** The genders of the groups are distributed homogeneously.

Group	*N*	Female	Male
Attack MS	5	3	2
Stable MS	14	8	6

There is no statistically significant difference between the mean ages of the groups (*p* = 0.701).

There is no statistically significant difference in the gender distribution of the groups (*p* = 0.964).

### 3.2 fMRI Analysis Findings

The regions showing significant functional connectivity in the attack and stabil RMMS patient groups evaluated in the study are given in Tables 3, 4, 5, 6, 7.

**TABLE 3:** Clustering findings of areas (voxels) showing significantly higher activity in MS patients in the relapse phase for the 3rd component (C3), highlighting the left fusiform cortex.

Cluster index	Voxels	Max	COG X (vox)	COG Y (vox)	COG Z (vox)	COPE‐max X (vox)	COPE‐max Y (vox)	COPE‐max Z (vox)	COPE‐mean
Left fusiform cortex	38	0.979	65	48.7	23	65	48	22	0.964

**FIGURE 3:**
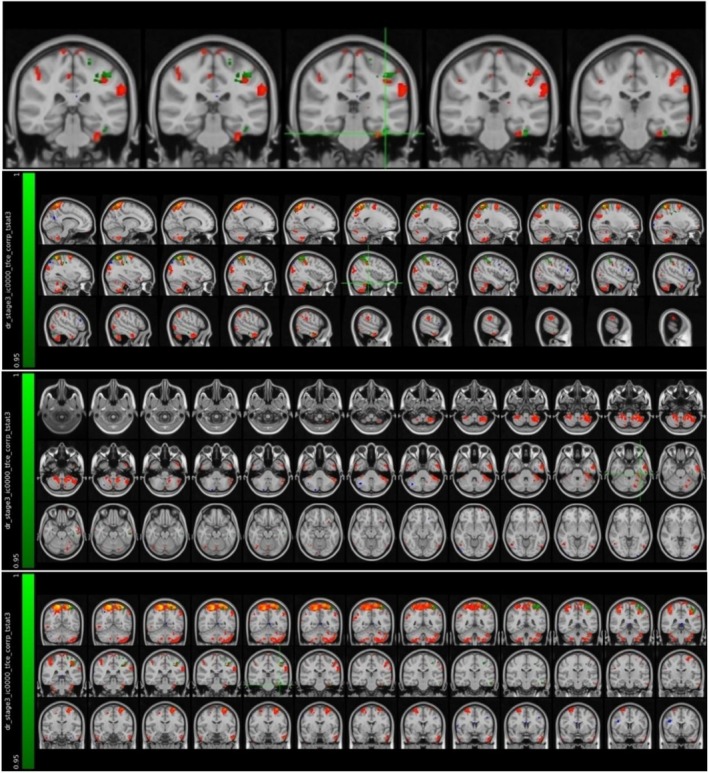
Voxels with activation in the left fusiform cortex.

It was found that the MS patient group in the attack period showed higher activation in the left fusiform cortex compared to the MS patient group in the stable period (Figure 3 and Table 3).

**FIGURE 4:**
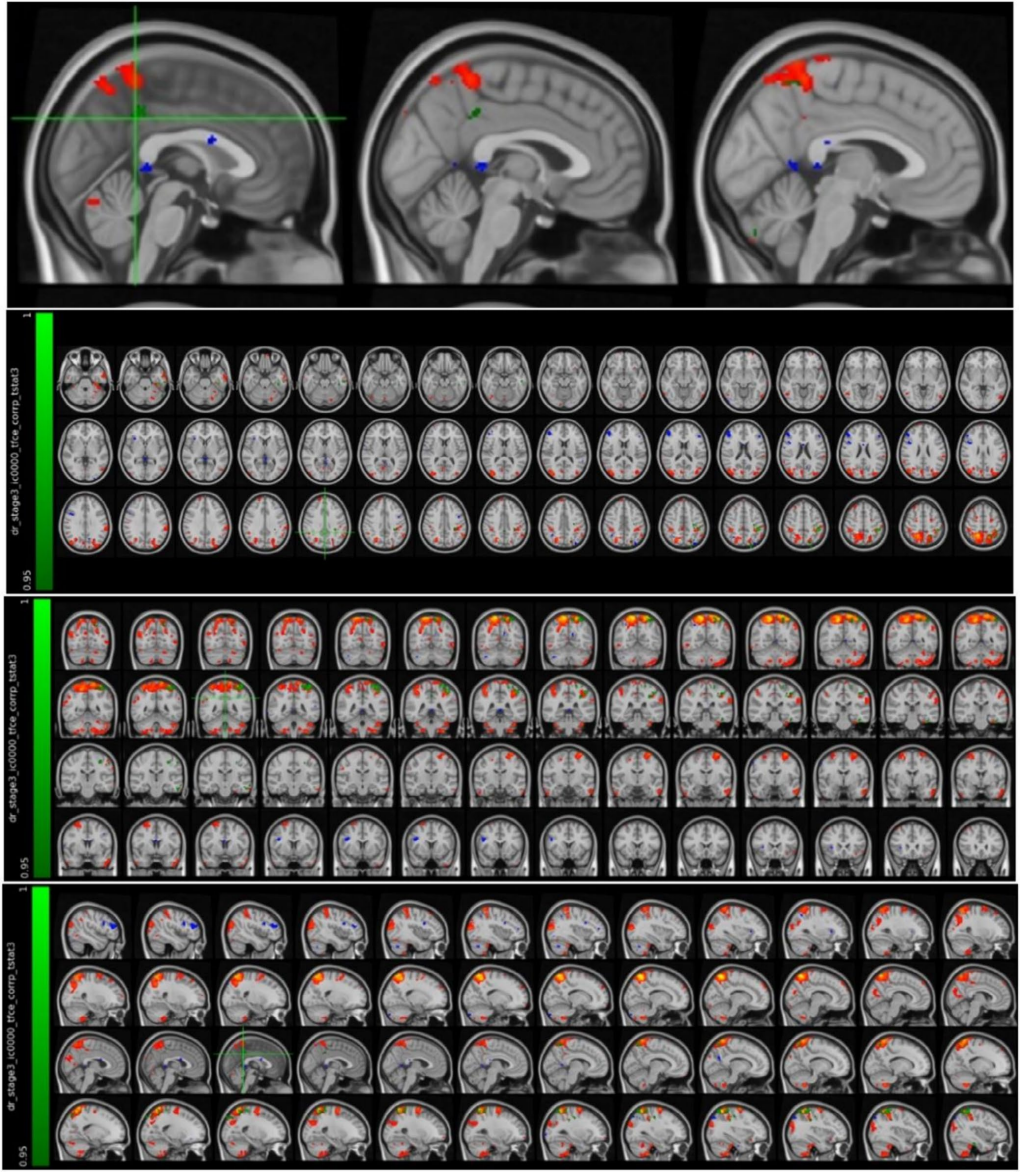
Voxels with activation in the left posterior cingulate cortex and precuneus region.

It was found that the MS patient group in the attack period showed higher activation in the left posterior cingulate cortex and precuneus region compared to the MS patient group in the stable period (Figure 4 and Table 4).

**TABLE 4:** Clustering findings of areas (voxels) showing significantly higher activity in MS patients in the relapse phase for the 3rd component (C3), highlighting the left posterior cingulate cortex and precuneus.

Cluster index	Voxels	Max	COG X (vox)	COG Y (vox)	COG Z (vox)	COPE‐max X (vox)	COPE‐max Y (vox)	COPE‐max Z (vox)	COPE‐mean
Left posterior cingulate cortex and precuneus	26	0.957	45.2	41.5	0.957	45	40	54	0.954

**FIGURE 5:**
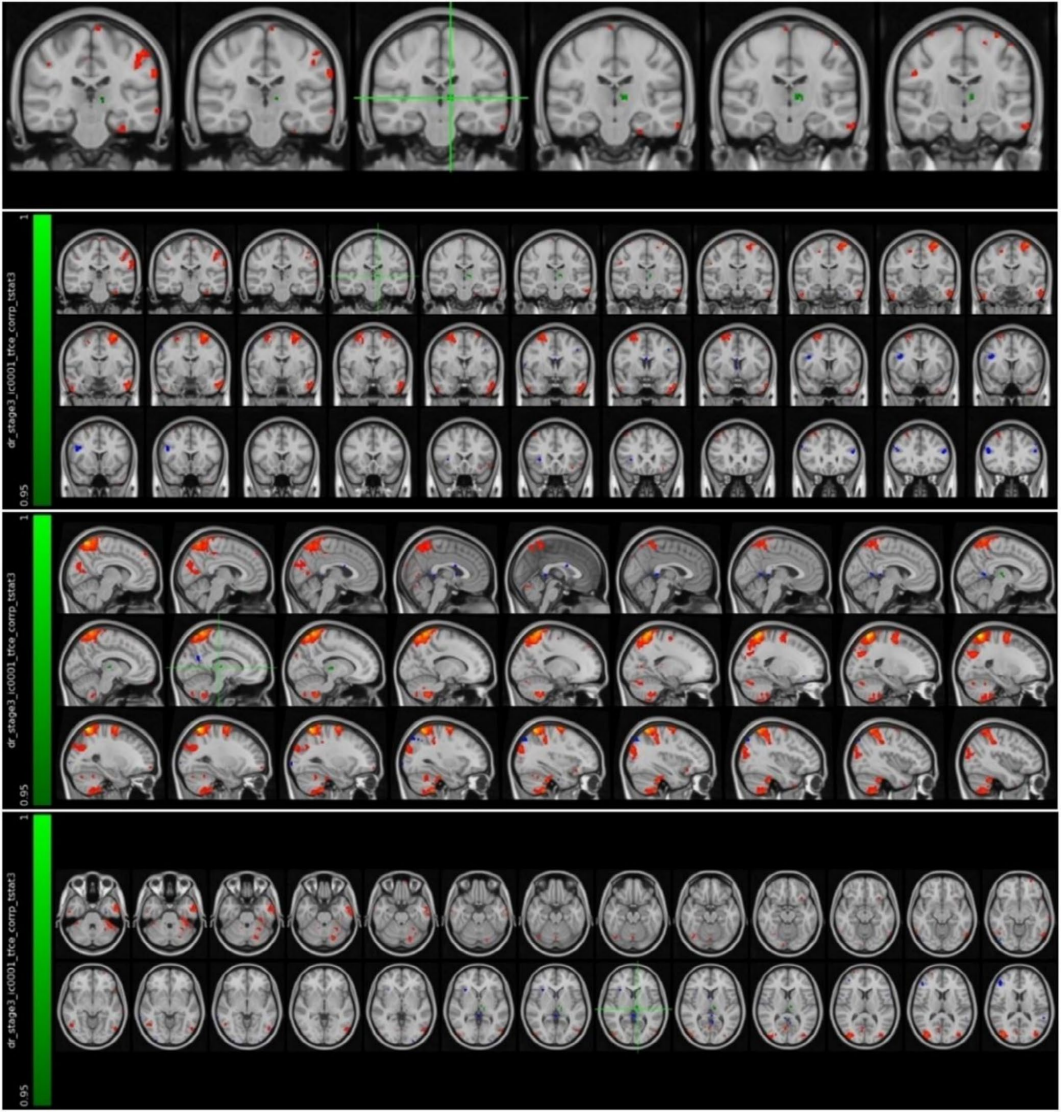
Voxels with activation in the left thalamus.

It was found that the MS patient group in the attack period showed higher activation in the left thalamus compared to the MS patient group in the stable period (Figure 5 and Table 5).

**TABLE 5:** Clustering findings of areas (voxels) showing significantly higher activity in MS patients in the relapse phase for the 3rd component (C3), highlighting the left thalamus.

Cluster index	Voxels	Max	COG X (vox)	COG Y (vox)	COG Z (vox)	COPE‐max X (vox)	COPE‐max Y (vox)	COPE‐max Z (vox)	COPE‐mean
Left thalamus	45	0.975	49.6	53.3	38.2	50	52	38	0.962

**FIGURE 6:**
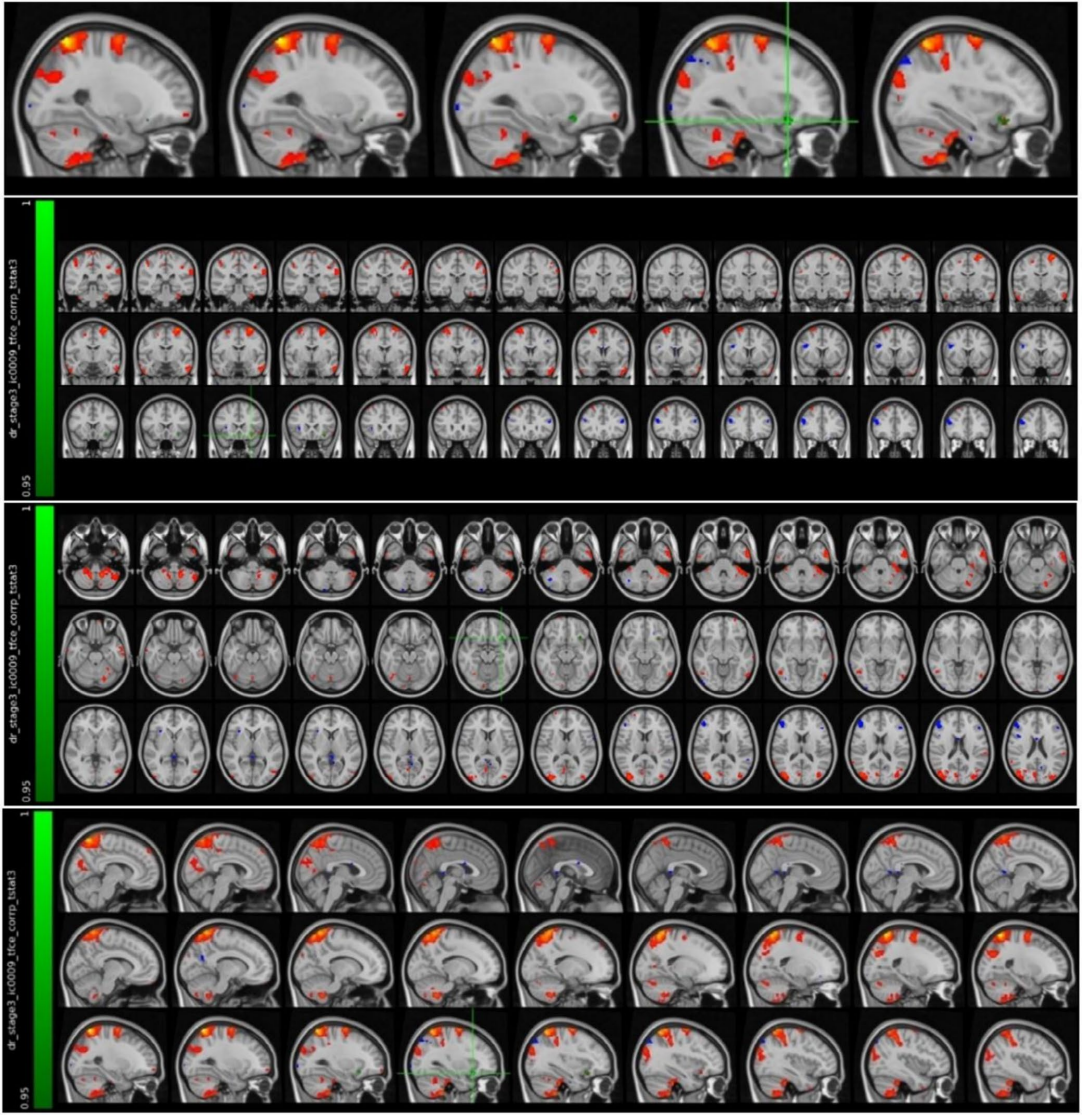
Voxels with activation in the left orbitofrontal cortex.

It was found that the MS patient group in the attack period showed higher activation in the left orbitofrontal cortex compared to the MS patient group in the stable period (Figure 6 and Table 6).

**TABLE 6:** Clustering findings of areas (voxels) showing significantly higher activity in MS patients in the relapse phase for the 3rd component (C3), highlighting the left orbitofrontal cortex.

Cluster index	Voxels	Max	COG X (vox)	COG Y (vox)	COG Z (vox)	COPE‐max X (vox)	COPE‐max Y (vox)	COPE‐max Z (vox)	COPE‐mean
Left orbitofrontal cortex	37	0.971	60.1	73	28.9	60	73	28	0.96

**FIGURE 7:**
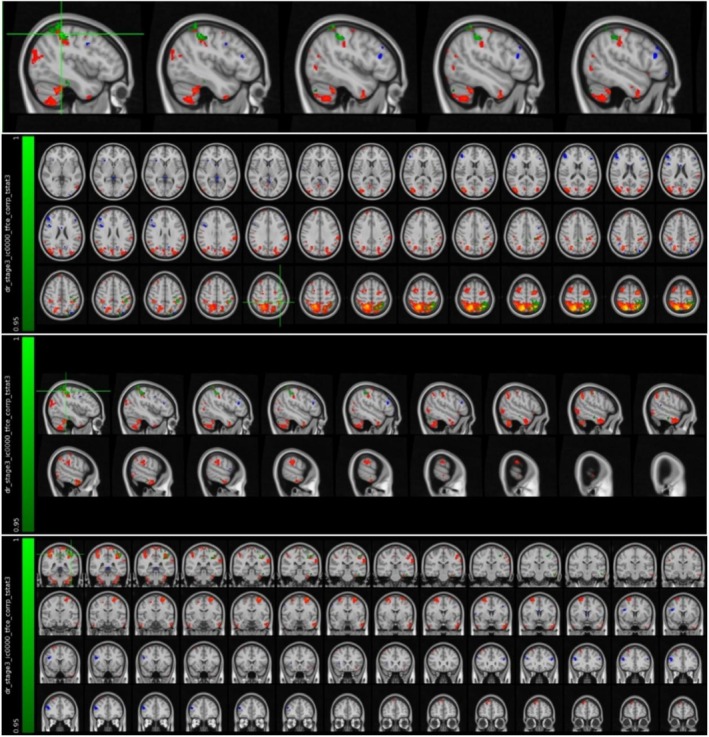
Voxels with activation in the left parietal lobe and supramarginal gyrus.

It was found that the MS patient group in the attack period showed higher activation in the left parietal lobe and supramarginal gyrus compared to the MS patient group in the stable period (Figure 7 and Table 7).

**TABLE 7:** Clustering findings of areas (voxels) showing significantly higher activity in MS patients in the relapse phase for the 3rd component (C3), highlighting the left parietal lobe and supramarginal gyrus.

Cluster index	Voxels	Max	COG X (vox)	COG Y (vox)	COG Z (vox)	COPE‐max X (vox)	COPE‐max Y (vox)	COPE‐max Z (vox)	COPE‐mean
Left parietal lobe and supramarginal gyrus	800	0.99	62	39.1	66.3	66	43	63	0.963

## 4 Discussion

Studies in the field of psychoneuroimmunology show that the CNS, autonomic nervous system, endocrine system, and immune systems are interrelated and affect each other through complex biochemical pathways [31, 32]. The aim of this study is to correlate functional connectivity differences in the CNS of MS patients with the immune system using fMRI.

When looking at the studies in the literature, it is clearly seen that the fusiform cortex is one of the cortical structures affected in MS disease. Studies have shown increased activation in bilateral fusiform cortex during object and human face recognition [33, 34]. In volumetric measurements performed on MS patients and healthy controls, significant atrophy was detected in the fusiform cortex in the patient group [35]. In another study aiming to reveal the relationship between the amount of cortical thickness, lesion size, and number of attacks in RRMS patients, it was stated that fusiform cortex thickness was negatively affected by these parameters [36]. The cortical thickness patterns of MS patients were examined, and it was revealed that the left fusiform cortex thickness differed significantly compared to healthy controls [37]. It was stated that in patients with weakened immune systems due to human cytomegalovirus (CMV) infection, the increase in IgG levels caused by this virus was parallel to the decrease in left fusiform cortex volume. In the same study, they stated that it was not clear whether this volumetric decrease was due to the susceptibility of the fusiform cortex to viral infections [38]. In another study evaluating T‐cells and cortical thickness in order to evaluate the neuroimmunological status of bipolar patients, a negative correlation was revealed between peripheral T‐cell level and left fusiform cortex thickness [39]. Studies correlating increased proinflammatory markers with volumetric decreases in the fusiform gyrus have taken their place in the literature in recent years [40, 41]. In another study evaluating the relationship between low brain volumes and high levels of anti‐inflammatory markers in the blood, a negative correlation was found between the levels of sTNF‐R1, an immunoregulatory cytokine, and the volumes of 10 brain regions, including the fusiform gyrus [40]. More recently, large‐scale RS‐fMRI repositories such as the Italian Neuroimaging Network Initiative (INNI) have confirmed reproducible network‐level alterations in MS, including visual network and fusiform‐related regions [42]. In addition, systematic reviews covering RS‐fMRI studies emphasize heterogeneous but consistent patterns of Default Mode Network (DMN) and visual network disruption linked to cognitive impairment [43]. In our study, we also found increased neuronal activation in the left fusiform cortex region of MS patients in the relapsing phase, whose inflammatory states increased compared to MS patients not in the relapsing phase. We believe that this finding in our study can be interpreted as parallel to volumetric and functional studies indicating the status of the left fusiform cortex in the inflammatory process. The fusiform cortex is associated with high‐level visual processing, and its increased connectivity may indicate compensatory mechanisms supporting sensory integration during inflammatory activity. Both the studies in the literature and the data obtained from our study raise the question of whether the fusiform cortex is a structure that is sensitive enough to lose volume in the event of peripheral inflammation or whether it is a cortical structure that produces an immune response by increasing activation in response to inflammation.

In a study evaluating the local gyrus index, which shows the amount of tortuosity of the cortical gyrus, it was found that there was a decrease in this index value in the left posterior cingulate cortex in RRMS patients. Again, in the same study, it was stated that the increase in the number of attacks was parallel to the decrease in the volumes of the left precuneus region and the right posterior cingulate cortex [36]. In the study evaluating the anatomical pattern of changes in cortical thickness in MS patients, it was revealed that there was a volumetric decrease in the bilateral posterior cingulate cortices of the patients, while the bilateral anterior cingulate cortex was preserved [44].

It was stated that the volumetric pattern displayed by the precuneus, which plays a primary role in the visual processing system and is one of the default mode network (DMN) centers, especially on the right side, is negatively correlated with T‐cell levels, indicating a relationship between the precuneus and the immune system [41]. Converging with our findings, recent RS‐fMRI studies report altered DMN connectivity, particularly involving the precuneus and posterior cingulate cortex, and link these changes to cognitive deficits in MS [45]. Longitudinal data in the EAE animal model further demonstrate that hyperconnectivity during inflammatory phases is followed by network decline, suggesting a temporal dynamic relevant to relapse–remission differences [46]. In our study, we found that MS patients in the relapsing period had more activation in the posterior cingulate cortex and left precuneus region on the left side in resting‐state fMRI data compared to MS patients who were not in the relapsing period. This finding is parallel to the studies in the literature. We believe that these data support the information that especially the posterior part of the cingulate cortex and the precuneus play a role in the inflammatory period in RRMS patients. Both regions are known hubs of the default mode network and are strongly linked to cognitive processes and immune signaling, suggesting that increased connectivity here may reflect heightened brain–immune system interaction during relapse.

Demyelination lesions in the thalamus are important findings in neuropathological studies and are known to originate from activated innate immune cells [47]. It has been concluded that this activation, especially seen in the thalamus, may be an inflammatory response to inflammation in the white matter structures extending to the thalamus or to neurodegeneration in the thalamus itself [48]. It is reported that there is a decrease in left thalamus volumes in the SPMS patient group in their study in which they made volumetric comparisons of the healthy control group and PPMS, SPMS, and RRMS patients [49].

It is compared the inflammatory profiles of the cerebrospinal fluids of RRMS patients and the volumes of brain regions affected in the early stages of the disease and adjacent to the ventricles (thalamus, hippocampus, and cerebellum) and other regions for control purposes (putamen, globus pallidus), and found statistically significant results in the gray matter surface areas of the structures adjacent to the ventricles [50]. They attributed this result to anatomical proximity, assuming that the reason for this was the inflammatory markers in the CSF of the ventricles. There are studies in the literature showing that CSF contents such as neurofilament light chain and parvalbumin, which are markers of neurodegeneration, are related to neuronal loss in the thalamus. Parvalbumin, which is a GABAergic neuronal degeneration marker found in the CSF, has been associated with gray matter atrophy. In this study, a significant link was found between the inherited immune activity and main inflammatory factors (fibrinogen, IL‐2, IL‐10, sTNF‐R1) in the CSF and increased microglial density in the thalamus in the MS group. Recent RS‐fMRI research has extended this to functional measures, showing that altered thalamic connectivity is strongly associated with cognitive impairment and impaired network controllability in MS patients [51].

The studies have attributed the thalamus to be one of the main structures affected in MS disease; its central location in the brain, its being affected by CSF due to its proximity to the ventricles, and its undertaking of functional tasks with many cortical structures and subcenters in the brain through numerous connection pathways [52]. When we evaluated the resting‐state fMRI images in our study, we found that there was increased functional connectivity in the left thalamus region of MS patients in the attack period compared to patients not in the attack period. We believe that this finding is similar to other studies in the literature that evaluated the thalamus and MS disease with different methods. Our questions about whether the thalamus activation increase in the attack period is a role that causes the inflammatory period of MS disease or whether it plays a protective role during this period are consistent with the findings and hypotheses in the literature. Given the thalamus's central role in sensory integration and its previously reported vulnerability to inflammatory processes in MS, this finding highlights its potential involvement in mediating immune‐related changes.

A negative correlation is found between the increase in CRP levels, which is an indicator of inflammation, and the decrease in the volumes of the right and left orbitofrontal cortex in their studies in bipolar patients [53]. It has been stated that the orbitofrontal cortex is associated with an increase in receptors that are effective in the regulation of neurotransmitter systems and immune responses, and a decrease in receptors related to intracellular transport, transcription regulation, and DNA repair [54]. It is stated that lateral orbitofrontal cortex thickness was reduced in HIV‐positive individuals with high peripheral inflammatory markers, and that this sheds light on the motor dysfunctions seen in these individuals. It is reported that the lateral part of the orbitofrontal cortex was affected by immune responses, and further studies were needed to confirm this finding [55]. It is demonstrated that brain regions belonging to the frontal cortex, such as the orbitofrontal cortex and medial prefrontal cortex, are associated with peripheral immune responses such as redistribution of lymphocytes and cardiovascular and neuroendocrine activities that regulate changes in immune functions [56]. In addition, treatment‐sensitive RS‐fMRI studies have shown modulation of orbitofrontal connectivity following disease‐modifying interventions, suggesting that inflammatory and therapeutic status should be considered when interpreting relapse‐related changes [57]. In our study, we found that there was more functional connectivity in the left orbitofrontal cortex in the MS group in the attack period compared to the group not in the attack period. We believe that this finding is parallel to the literature studies indicating immune response and orbitofrontal cortex, which are more common in patients in the attack period. The orbitofrontal cortex is functionally associated with decision‐making and emotional regulation, but also with stress and immune responses, supporting the hypothesis that cortical regions contribute to the brain's immunological adaptations.

It is reported that RRMS and SPMS patients developed significant atrophy in the supramarginal gyrus of the parietal lobe compared to the control group. In the same study, it was reported that supramarginal gyrus atrophy was positively correlated with EDSS values [49]. The susceptibility of the supramarginal gyrus to neuropathological processes in Alzheimer's patients is based on the thesis that it is a part of the heteromodel cortex that undergoes significant myelination during development [58]. Recent resting‐state studies also indicate that parietal network connectivity changes, including supramarginal regions, are related to clinical disability measures such as the Symbol Digit Modalities Test (SDMT) and EDSS [45]. In our study, we found that the left supramarginal gyrus and adjacent parietal lobe areas showed greater functional connectivity and activation in MS patients in the relapsing period than in those not in the relapsing period. We believe that this finding is parallel to the findings in the literature regarding the supramarginal gyrus in both MS patients and other neurodegenerative patients. These regions are involved in sensory‐motor integration and attention, and their increased activation may represent compensatory reorganization to limit the impact of inflammatory damage on clinical functioning.

When the studies evaluating the relationship between MS and other neurodegenerative diseases and inflammation and the findings of our study are examined, it is generally observed that functional connectivity and atrophy develop on the left side of the brain. It is known that brain asymmetry, revealed by anatomical, neurochemical, and functional data, plays a role in the regulation of physiological mechanisms including the immune system. The effect of brain asymmetry on immune functional activity and cytokine production has been demonstrated. It is stated that T‐cells are suppressed in left‐sided brain lesions and activated in right‐sided lesions [59, 60]. In our study, MS patients in the relapse period mostly show increased functional connectivity on the left side. This finding led us to think that the immune system shows lateralization in the brain. The patients in the attack phase consistently demonstrated increased connectivity in gray matter regions linked to cognition, sensory integration, and immune‐related signaling. This suggests that the CNS responds to acute inflammation by engaging compensatory functional networks, which may serve to buffer clinical manifestations of disease activity.

## 5 Limitations

There are several limitations of this study. First, the relapse group included only a small number of patients (*n* = 5), which limits the statistical power and generalizability of our findings. While standardized fMRI preprocessing and analysis methods were applied to mitigate some risks, the results should be interpreted with caution. Second, although EDSS scores were standardized and all patients were in the early stage of MS with mild symptoms (EDSS range 1.5–3.5), we did not have sufficient data on cognitive performance, detailed immunological measures, or blood/CSF markers to correlate with functional connectivity changes. Finally, the acquisition of fMRI images from different patient groups in the relapse and stable phases can be considered a methodological limitation of our study. Incorporating these parameters in future studies, along with a healthy control group and task‐based fMRI paradigms, could provide a more comprehensive understanding of the clinical relevance of these findings and further test the hypotheses generated in this study.

## 6 Conclusion

Our findings revealed alterations in functional connectivity in several gray matter regions, including the fusiform cortex, cingulate, precuneus, thalamus, orbitofrontal cortex, and supramarginal gyrus, particularly in patients during the relapsing phase. These results are generally consistent with previous volumetric and functional studies, which have demonstrated structural changes and altered connectivity patterns in similar cortical and subcortical regions [33, 34, 35, 36, 37, 49].

Specifically, increased functional connectivity in the left fusiform cortex and posterior cingulate cortex observed during relapses may reflect the involvement of these regions in inflammatory processes. However, it remains unclear whether these changes represent compensatory mechanisms or indicate sensitivity of these structures to peripheral inflammation, raising the hypothesis that these regions may either be particularly vulnerable to immune‐related processes or actively participate in immune responses. Similarly, increased connectivity in the left thalamus and orbitofrontal cortex during attacks may suggest an interaction between immune activity and regional brain function, consistent with prior neuroimmunological evidence [41, 47, 48, 55].

In conclusion, our results suggest that functional connectivity differences in specific gray matter regions during inflammatory attacks may precede structural atrophy observed in later stages of MS. These findings support the hypothesis that certain brain regions may play a central role in immune‐related processes in early‐stage MS. Nevertheless, further studies with larger cohorts, comprehensive clinical assessments, and multimodal neuroimaging are required to validate and extend these observations.

## Author Contributions

**Gamze Ansen:** conceptualization, methodology, data curation, formal analysis, writing – original draft, review and editing. **Ali Behram Salar:** data curation, formal analysis, experimental procedures. **Abdulkadir Ermis:** methodology, data curation, experimental procedures. **Erkingul Birday:** methodology, data curation. **Lutfu Hanoglu:** conceptualization, methodology, supervision. **Bayram Ufuk Sakul:** conceptualization, supervision, review.

## Conflicts of Interest

The authors declare no conflicts of interest.

## Data Availability

The data that support the findings of this study are available from the corresponding author upon reasonable request.
